# Journal Retraction Rates and Citation Metrics: An Ouroboric Association?

**DOI:** 10.7759/cureus.11542

**Published:** 2020-11-18

**Authors:** Amrutha B Nagella, Venkatesh S Madhugiri

**Affiliations:** 1 Anesthesiology and Critical Care, Bangalore Medical College and Research Institute, Bangalore, IND; 2 Neurosurgery, National Institute of Mental Health and Neurosciences, Bangalore, IND

**Keywords:** retraction index, retraction rate, impact factor, h-index, anesthesia, neurosurgery, citation metrics, retraction

## Abstract

Introduction

Retraction of published papers has a far-reaching impact on the scientific world, especially if the retracted papers were published in high-impact journals. Although it has been noted that the retraction rates of journals correlated with their citation metrics, no conclusive data were available for most clinical specialties. In this study, we determined the retraction rate for anesthesia and two comparison groups (neurosurgery and high impact clinical journals). We then studied the correlation of the retraction rate with citation metrics.

Methods

We generated a list of all anesthesia journals that were indexed in the National Library of Medicine database. We obtained the number of papers published in each journal as well as the number of papers retracted from each. We also collated the Impact Factor® and H-index of each journal. The same methodology was followed for neurosurgery and high impact clinical journals. We then studied the correlations between the retraction rate and citation metrics of each journal.

Results

The retraction index was 2.59 for anesthesiology, 0.66 for neurosurgery and 0.75 for the high-impact clinical journals group. The retraction rate did not correlate with the citation metrics. However, the number of papers published in each journal and the absolute number of retractions showed a positive correlation with the citation metrics. The H-index showed stronger correlations with these parameters than the Impact factor.

Conclusions

The number of retractions increased in proportion to both the number of papers published in a journal and the citation metrics of that journal.

## Introduction

Retraction of published papers is an inevitable part of the scientific process and reflects the self-correcting nature of science. However, as the volume of scientific literature increases exponentially, the number of retractions has also been increasing [1]. The increasing number of retractions could reflect better engagement of the scientific community with the process of post-publication review. However, it could also be due to an increased rate of malpractice, owing to the “publish or perish” pressure that is now ubiquitous in science. Startling examples of malpractice that led to immediate real-world harm were seen during the ongoing COVID pandemic. A particularly egregious instance is that of two papers by the same lead authors, that were published in the Lancet and the New England Journal of Medicine (NEJM), respectively [2,3]. Both of these papers appear to have been based on completely fraudulent data [4,5]. The effect of these papers was to temporarily halt the hydroxychloroquine arm of the multi-national SOLIDARITY trial being run by the World Health Organization, which was then restarted once the papers were retracted. The COVID pandemic has led to a rush to publish any science related to the novel coronavirus. This has often led to compromised and inadequate pre-publication peer review [6]. The number of COVID-related papers available on PubMed Central (as of November 6, 2020) was 74993; 38 of these papers have been retracted currently, yielding a retraction rate of 0.05% [7]. This is much higher than the rate of retractions for the life sciences as a whole, which was 0.01% [8]. The consequences of the dissemination of false information via compromised papers are all too well known. The effects of the (now discredited) paper published by Wakefield et al are still felt the world over, in the form of a burgeoning anti-vaccine movement [9,10].

The exact impact of scientific papers is difficult to quantify. Citation metrics are imperfect markers to assess the impact of journals or published papers on science and the community. However, it cannot be gainsaid that the papers published in highly cited journals are more widely disseminated than those published in lower cited (“low impact”) journals. Several studies have found that the retraction rates for journals correlate with the Impact Factor^(R)^ (ImpFac), and thus, rates of retraction are higher for higher cited journals [11,12]. This would imply that higher cited journals publish more compromised papers that eventually get retracted, but are also more widely disseminated prior to retraction. However, this association has not been reported consistently and some studies have found either no association or a negative association between the retraction rates in a scientific discipline and the ImpFac of journals in that discipline [13,14].

In this study, we examined if the number and rates of retraction (represented by the “retraction index”) correlated with the citation metrics (ImpFac and H-index) of journals in the field of anesthesiology. In order to validate the results, we also generated two comparison groups and ran the same analyses across the comparison groups.

## Materials and methods

Anesthesia

We first identified all PubMed indexed anesthesia journals from the National Library of Medicine database [15]. The database was searched using various search strings (anesthesia, anesthesiology, etc.). The results from the searches using these strings were collated and duplicates eliminated, to generate a list of all indexed journals for anesthesia as a broad specialty. 

We then searched the Retraction Watch database using the name of each journal from the preceding list, to identify the number of retractions from each journal (R) over the past 10 years (2010-2020, both inclusive) [16]. The R number for each journal was also cross-checked by searching PubMed using the name of the respective journal as a search string and with the retraction filters applied [17]. For instance, the search strategy on PubMed to identify R for the Journal of Anesthesia would be - 

"j anesth"[Journal] AND ((Retracted Publication[sb] OR Retraction of Publication[sb]) AND ("2010/01/01"[PDAT] : "2020/12/31"[PDAT]))

The numbers of retractions in the past 10 years for each anesthesia journal were collated. The number of indexed papers published in each journal over the past 10 years (N) was determined by searching PubMed using the time filters. For example, the search strategy to determine N for the Journal of Anesthesia would be -

"j anesth"[Journal] AND ("2010/01/01"[PDAT] : "2020/12/31"[PDAT])

The retraction index for a journal was computed using the following formula [12]:

$RIi=\left ( \frac{Ri}{Ni} \right )\times 1000$

Thus, the RI basically denotes the number of papers retracted for every 1000 papers published by the journal over the defined time period. Thus, the RI was computed for each anesthesia journal. The median RI for anesthesia as a specialty (and anesthesia journals as a group) was then computed.

It should be noted that the preceding methodology studies retractions of anesthesia papers published in “pure” anesthesia journals. Anesthesia papers published in other specialty journals or in general medical journals (such as the NEJM) would not be included in this dataset, since the retraction rates for those journals would be driven by other specialties.

The ImpFac of each journal was obtained from the Journal Citation Reports (JCR) 2020 list (Clarivariate Analytics). For those journals that were not included in the JCR list, the citations-per-document (over a two-year period) statistic was obtained from the Scimago site [18]. This statistic is computed using a methodology that is nearly congruent to the ImpFac calculation process. The H-index for each journal was obtained from the Scimago website. 

Spearman’s rank order correlation was used to evaluate for any association between the RI and ImpFac or H-index. Similar correlation analyses were also carried out between the raw number of retracted papers (R) and the ImpFac and H-index. All analyses were carried out on Stata (v14, Stata Corp, College Station, TX) and Microsoft Excel (v16.16.22, Microsoft Corp, WA).

Comparison groups

In order to validate the methodology described above, we generated two comparison groups. For the first comparator group, we elected to study retractions across neurosurgery journals. The same methodology as employed for anesthesia was used to generate the list of neurosurgery journals and to determine R, N, and RI values for each neurosurgery journal. 

The second comparator group was generated from the JCR 2020 list. We selected all journals pertaining to clinical disciplines from among the top 200 journals in the JCR. Journals that were common between the anesthesia and neurosurgery groups and this group were preferentially included in the respective specialty group. Journals that exclusively published reviews were not included. Once the list of the highest impact clinical journals was parsed from the top 200 journals on the JCR, the subsequent methodology to identify the R, N and RI was the same as previously described.

## Results

Thirty-two anesthesia journals, 23 neurosurgery journals, and 41 high ImpFac clinical journals (HICJs) from the JCR 2020 were finally included in the analysis.

Anesthesia journals

The cumulative number of retractions over the past 10 years, across the 32 anesthesia journals included in the analysis (R) was 334 (Table 1). The number of indexed papers published across these journals over the same period (N) was 70286. The overall retraction index for anesthesia journals for the period 2010-2020 was 2.59 (range: 0-17.69). The overall RI for anesthesia was 4.75. 

**Table 1:** Anesthesia journals included in the analysis and their attributes The journals are arranged in descending order of their retraction indices (RI). R = number of retracted papers; N = the total number of indexed papers published in these journals over the past 10 years; RI = retraction index.

Journal	Impact Factor	H index	R	N	RI
European Journal of Anesthesiology	4.5	73	32	1809	17.69
Can J Anesth	3.779	92	39	2244	17.38
Saudi Journal of Anesthesia	1.287	22	18	1396	12.89
Anesthesia & Analgesia	4.305	195	59	5837	10.11
British Journal of Anaesthesia	6.88	170	44	4368	10.07
JA Clinical Reports	0	1	3	376	7.98
Acta Anesthesiologica Scandinavica	2.05	103	15	1958	7.66
Journal of Anesthesia	1.628	42	13	1843	7.05
Anesthesiol Intensivmed Notfallmed Schmerzther	0.507	26	6	933	6.43
Annals of Cardiac Anesthesia	1.26	24	5	1080	4.63
Anesthesia	5.739	111	17	3732	4.56
Der Anesthesist	1.095	42	6	1392	4.31
Journal of Dental Anesthesia and Pain Medicine			1	259	3.86
Anesthesia and Intensive Care	1.539	61	6	1562	3.84
Pediatric Anesthesia	2.311	79	9	2359	3.82
Anesthesia, Essays and Researches			3	1131	2.65
Journal of Cardiothoracic and Vascular Anesthesia	2.258	80	11	4364	2.52
Journal of Clinical Anesthesia	6.039	68	7	2853	2.45
Intensive Care Medicine	17.679	187	10	4149	2.41
Regional Anesthesia and Pain Medicine	7.015	103	4	1770	2.26
International Journal of Obstetric Anesthesia	1.895	51	2	1003	1.99
Anesthesiology	7.067	225	9	4551	1.98
Anesthesiology and Pain Medicine	1.176	23	1	666	1.50
Journal of Anesthesiology, Clinical Pharmacology	0.039	28	2	1517	1.32
Egyptian Journal of Anesthesia	0.365	10	2	1566	1.28
Minerva anestesiologica	2.498	56	2	2299	0.87
Journal of Clinical Monitoring and Computing	2.108	48	1	1308	0.76
BMC Anesthesiology	1.695	36	1	1412	0.71
The Laryngoscope	2.465	142	4	6373	0.63
Korean Journal of Anesthesiology	2.094	26	1	1703	0.59
Indian Journal of Anesthesia	1.412	26	1	2214	0.45
Acta Anesthesiologica Taiwanica	0.559	25	0	259	0.00

**Figure 1:**
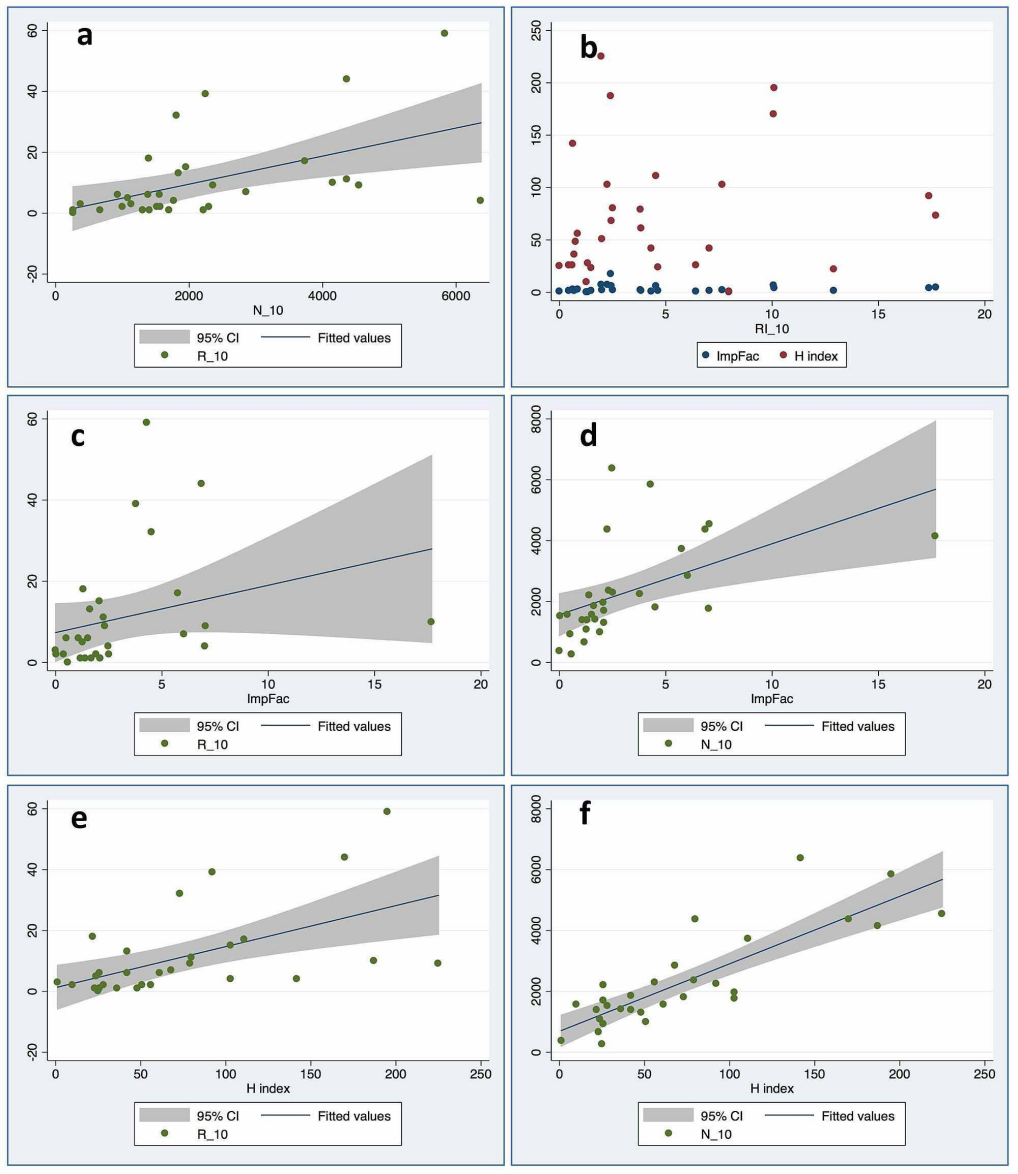
Graphical depiction of the relationship between pairs of variables from the analysis The graphs are scattered plots, the line depicts the regression line and the shaded area depicts the 95% CI of the predicted values. a = R vs N; b = RI vs Impact Factor and H-index; c = Impact factor vs R; d = Impact factor vs N; e = H-index vs R; f = H-index vs N.

The number of papers retracted from anesthesia journals over the period 2010-2020 (R) correlated positively with the number of papers published during the same period (N) (Table 2). The RI did not correlate with the ImpFac; however, both R and N correlated positively with the ImpFac (Figure 1). Similar results were obtained when the H-index was used as the citation metric. The RI did not correlate with the H-index but both R and N correlated positively with the H-index (Table 2). The strength of the correlation between R and ImpFac was not different from the strength of the correlation between R and H-Index (z=-1.206, p=0.114). Likewise, the H-index and ImpFac correlated equally strongly with N (z=-0.862, p=0.194). 

**Table 2:** The results of Spearman’s correlation analysis between pairs of variables for anesthesia, neurosurgery and high impact clinical journals R = number of retracted papers; N = number of indexed papers published over the same period; RI = retraction index; ImpFac = Impact factor.

Correlation pair	Spearman’s r	p value
Anesthesia		
R-N	0.6	0.0003
RI- ImpFac	0.136	0.474
R- ImpFac	0.496	0.005
N- ImpFac	0.774	<0.0001
RI- H-index	0.21	0.266
R- H-index	0.594	0.0005
N- H-index	0.822	<0.001
Neurosurgery		
R-N	0.304	0.159
RI- ImpFac	0.311	0.159
R- ImpFac	0.514	0.014
N- ImpFac	0.134	0.553
RI- H-index	-0.21	0.35
R- H-index	0.511	0.015
N- H-index	0.703	0.0003
High Impact Clinical Journals		
R-N	0.775	<0.0001
RI- ImpFac	0.218	0.171
R- ImpFac	0.409	0.008
N- ImpFac	0.45	0.003
RI- H-index	0.469	0.002
R- H-index	0.773	<0.0001
N- H-index	0.83	<0.0001

Comparator group: neurosurgery

The R for the period 2010-2020 across the 23 neurosurgery journals was 57 and N was 71939. (Table 3) The median RI for neurosurgery journals was 0.66 (range 0.2-4.3) and the overall RI was 0.79. Neither the ImpFac nor the H-index showed a significant correlation with the RI. A positive correlation was noted between R and the ImpFac, but not between ImpFac and N. The H-index showed a positive correlation with both R and N (Table 2). 

**Table 3:** Neurosurgery journals included in the analysis and their attributes The journals are arranged in descending order of their retraction indices (RI). R = number of retracted papers; N = the total number of indexed papers published in these journals over the past 10 years; RI = retraction index.

Journal	ImpFac	H index	R	N	RI
Neuro-oncology	10.247	113	9	2076	4.33526012
J Spinal Disord Tech	3.3	79	2	654	3.05810398
Cureus			3	1259	2.38284353
Neurosurgical Focus	3.642	90	4	2026	1.97433366
J Neurooncol	3.267	111	6	3360	1.78571429
Asian Spine J	1.338	24	2	1189	1.68208579
Neuro-Chirurgie	1.289	28	1	706	1.41643059
J Neurosurg Sci	1.645	34	1	846	1.1820331
Acta Neurochir Suppl	3.076	65	1	998	1.00200401
Neurosurgical review	2.654	58	1	1102	0.90744102
J Neurosurg	3.968	200	4	5542	0.7217611
J Korean Neurosurg Soc	1.376	32	1	1510	0.66225166
Neurosurgery	4.853	192	3	5302	0.56582422
World neurosurgery	1.829	90	8	14241	0.5617583
Acta neurochirurgica	1.817	91	2	3736	0.53533191
Br J Neurosurg	1.29	62	1	2096	0.47709924
J Neuroint Surg	4.46	49	1	2449	0.40832993
J Neurosurg Spine	3.011	93	1	2521	0.39666799
Surgical Neurology International	0.963	30	1	2670	0.37453184
Spine	2.646	243	2	5688	0.35161744
The Spine Journal	3.191	102	1	3470	0.28818444
Child's nervous system	1.298	80	1	3610	0.27700831
European spine journal	2.458	128	1	4888	0.20458265

The H-index and ImpFac correlated equally strongly with R (z=-0.018, p=0.493). However, the correlation between the H-index and the number of papers published over the 10-year period studied (N) was much stronger (z=-3.345, p<0.0001).

Comparator group: high-impact factor clinical journals

The R for the HICJ group was 192 papers and N was 233632 (Table 4). The median RI for the HICJ group was 0.75 (range: 0-3.05) and the overall RI was 0.82. R correlated strongly with the total number of indexed papers published in the HICJs (N) (Table 2). There was no correlation between the RI and ImpFac but both R and N correlated positively with ImpFac. The H-index correlated strongly with the RI, R and N (Table 2).

**Table 4:** High impact clinical journals included in the analysis and their attributes The journals are arranged in descending order of their retraction indices (RI). R = number of retracted papers; N = the total number of indexed papers published in these journals over the past 10 years; RI = retraction index.

Journal	ImpFac	H index	R	N	RI
JAMA Psychiatry	17.471	352	6	1964	3.055
NATURE MEDICINE	36.13	524	7	3811	1.837
JAMA Pediatrics	13.946	172	5	2779	1.799
Lancet Diabetes Endocrinol	25.34	97	3	1705	1.760
BLOOD	17.543	448	20	12757	1.568
Cancer	292.278	292	9	6541	1.376
HEPATOLOGY	14.679	347	8	6022	1.328
AMERICAN JOURNAL OF RESPIRATORY AND CRITICAL CARE MEDICINE	17.452	359	9	6837	1.316
NATURE IMMUNOLOGY	20.479	368	3	2305	1.302
DIABETES CARE	16.019	346	7	5548	1.262
JAMA Internal Medicine	18.652	329	6	4861	1.234
EUROPEAN HEART JOURNAL	22.673	286	9	7363	1.222
JAMA	45.54	654	17	13931	1.220
JOURNAL OF THE AMERICAN COLLEGE OF CARDIOLOGY	20.589	419	12	9851	1.218
JOURNAL OF CLINICAL ONCOLOGY	32.956	525	10	8410	1.189
Ann Oncol	18.274	229	5	4840	1.033
CIRCULATION	23.603	593	9	9052	0.994
JOURNAL OF HEPATOLOGY	20.582	232	4	4411	0.907
JAMA Oncology	24.799	80	2	2445	0.818
EUROPEAN UROLOGY	17.947	204	4	5131	0.780
Alzheimers Dement	17.127	107	1	1327	0.754
Lancet	60.392	747	12	16104	0.745
ANNALS OF INTERNAL MEDICINE	21.317	376	5	7571	0.660
N Engl J Med	74.699	987	9	15252	0.590
Lancet Respir Med	25.094	93	1	2294	0.436
ANNALS OF THE RHEUMATIC DISEASES	16.102	228	2	4623	0.433
LANCET ONCOLOGY	33.752	305	2	5141	0.389
GASTROENTEROLOGY	17.373	386	2	6450	0.310
J Thorac Oncol	13.357	123	1	3444	0.290
BMJ-British Medical Journal	30.223	412	2	29792	0.067
World Psychiatry	40.595	81	0	941	0.000
LANCET NEUROLOGY	30.039	280	0	2536	0.000
Lancet Infect Dis	24.446	217	0	3975	0.000
GUT	19.819	279	0	3218	0.000
Lancet Psychiatry	16.209	65	0	2012	0.000
Lancet HIV	14.813	44	0	959	0.000
Lancet Gastroenterol Hepatol	14.789	35	0	810	0.000
JAMA Surgery	13.625	170	0	2775	0.000
JAMA Neurology	13.608	220	0	2475	0.000
Sci Immunol	13.44	35	0	447	0.000
Trends Immunol	13.422	218	0	922	0.000

The H-index correlated more strongly with R than the ImpFac (z=-3.095, p=0.001). The H-index also correlated more strongly with N than the ImpFac did (z=-3.583, p<0.0001). 

Multivariate analysis: number of retractions and retraction index

The median RI for anesthesia (2.59), the HICJs (0.75) and for neurosurgery (0.66) were significantly different (Kruskal Wallis test, H=29.88, p<0.0001). Anesthesia had the highest RI over the past 10 years. 

We performed multivariable regression to identify factors that predicted the RI. For anesthesia, none of the regressors, viz N (t=-0.95, p=0.35), ImpFac (t=-0.6, p=0.55) or H-index (t=1.23, p=0.23) could independently predict the RI. For neurosurgery, ImpFac (t=4.35, p<0.0001) independently predicted the RI. For the HICJ group, the H-index (t=2.35, p=0.024) independently predicted the RI.

We also performed multivariable regression to identify factors that predicted the absolute number of retractions (R). For anesthesia, none of the regressors, viz N (t=-0.02, p=0.98), ImpFac (t=-1.1, p=0.28) or H-index (t=1.95, p=0.06) could independently predict the R. For neurosurgery, ImpFac (t=5.13, p<0.0001) and N (t=4.44, p<0.0001) independently predicted the R. For the HICJ group, the H-index (t=3.7, p=0.001) independently predicted R.

## Discussion

Retractions in anesthesia

The rate of retractions (RI) varies by scientific discipline. Based on the available literature, the RI is 1.5 for genetics, 0.29 for nursing and 0.1 for the life sciences as a whole [8,14,19]. In the present study, the RI for the period 2010-2020 (inclusive) was 2.59 for anesthesiology, 0.66 for neurosurgery and 0.75 for the HICJ group. Thus, anesthesia appears to have a higher retraction rate than neurosurgery and the HICJ groups; the RI for anesthesia is also higher than that for the life sciences as a whole. Most retractions in anesthesia appear to be due to misconduct by authors, especially data fabrication and plagiarism [20,21]. These reasons for retraction are nearly congruent to those in other specialties [22-24]. 

However, there is one phenomenon that largely determines the retraction milieu for anesthesia journals. Retractions in anesthesia have been largely driven by a few researchers and groups, unlike in other disciplines. An analysis in 2018 found that 313 papers that were eligible for retraction (278 already retracted and 35 not retracted yet) could be attributed to just three anesthesia researchers/ practitioners [25]. Furthermore, when retractions in the corpus of scientific literature as a whole were considered, the top two authors with the highest number of individual retractions, were both from the field of anesthesiology [26]. This phenomenon of individuals driving the bulk of retractions in anesthesia skews the retraction index for the specialty. 

The journal with the highest rate of retractions in anesthesia was the European Journal of Anesthesiology (EJA), with an RI of 17.69 (Table 1). Of the 32 retractions from the EJA, eight could be linked to Boldt and 12 to Fujii. If the retractions attributable to these two authors were to be eliminated from the calculation, the RI for EJA would be 6.7 per 1000 papers. Another striking example of a few authors driving the retraction rates of anesthesia journals is that of the Canadian Journal of Anesthesia (CJA). Thirty-four of 39 papers retracted from the CJA were authored by Fujii and the remaining five by Boldt. The current RI for the CJA is 17.38 per 1000 papers published. Had the systematic fraud perpetrated by Fujii, Boldt and Scott Reuben not occurred, the RI for the CJA would be 0.

Thus, when the high rate of retractions in anesthesia is discussed, it is necessary to always present this caveat, so as to present the true impact of systematic fraud on medical science. Sadly, the kind of systematic fraud perpetrated in anesthesia now appears to have been detected in the field of obstetrics and gynecology as well and an investigation is currently ongoing [27]. This large set of fraudulent papers would inevitably alter the retraction milieu for obstetrics and gynecology. While it cannot be denied that post-publication scrutiny was responsible for detecting these instances of fraud, these examples also underscore the importance of rigorous pre-publication peer review so as to prevent the dissemination of fraudulent science and erosion of the public faith in the scientific process. Increasing the number of peer reviewers and assistant editors so as to reduce the workload on each, could be one measure towards this end. 

Retractions in highly- vs lower-cited journals

The issue of retractions from highly cited journals is an important one. It has been widely noticed that the highly cited journals retract more papers than lower cited ones. The reasons for this could be several. First, the papers published in high impact journals are read more widely and are likely scrutinized more extensively. Second, the data published in high impact journals is more likely to prompt a spate of replication studies; thus, results that have poor replicability or are fraudulent are more consistently detected. The third reason would indicate that at least part of the blame must be ascribed to the journals themselves. The tendency of the high impact journals to almost exclusively publish strongly positive studies could act as an inducement for data fabrication. Publication in such high impact journals is likely to lead to significant advancement of a researcher’s career; this could be another possible reason to publish compromised data in the high impact journals [12]. The positive association between the rate of retractions and the citation metrics is disturbing, since fraudulent or compromised data published in highly cited journals are widely disseminated and continue to be cited even after retraction [28]. 

In this study, R (but not RI) showed a positive correlation with the citation metrics, implying that the higher impact journals retracted more papers than the lower impact ones. However, there is another important nuance to be considered. Since higher visibility and scrutiny of the articles published in high impact journals leads to more retractions (vide supra), the gap between the number of retracted papers and the number of retractable papers is very small in high impact journals. This gap (retractable articles - retracted articles) is much higher in lower impact journals [11]. Moreover, visibility of the article also correlated with greater scrutiny and earlier retraction [11]. Thus, a higher rate of retractions in high-impact journals is in all likelihood, the result of a better post-publication review. The returns of publishing in highly-cited journals could act as an inducement to publish papers with strongly positive results, often based on compromised data. However, papers published in the high-impact journals are highly cited, and ipso facto, are also scrutinized more than their counterparts in lower-cited journals. This increased scrutiny possibly results in a higher number of retractions from the higher-cited journals, thus leading to an ouroboric relationship between the citation metrics and the number of retractions from journals.

All journals should therefore consider implementing measures to improve the visibility of the published papers so as to improve the process of post-publication review, such as automatic open access to papers after a specified lock-in period. Shifting the emphasis of journals from only publishing studies with positive results (and vanishingly small p values), towards a focus on the strength of the study protocols, rates of protocol adherence and rates of follow-up (for clinical studies), could, in itself, act as an incentive to publish honest data. Extensive plagiarism and reference checks, and mandatory data deposition are steps that could aid in the detection of fraud prior to publication rather than in a post hoc fashion [29]. 

Rate and number of retractions vs citation metrics

In this study, we found that the rate of retraction (as described by the RI) did not correlate with either the ImpFac or H-index but the absolute number of retractions (R) and the number of indexed papers published (N) over the past 10 years correlated positively with both H-index and ImpFac (Table 2). Thus, unsurprisingly, as a journal (and specialty) published more papers, more retractions were to be expected therein. Although it is widely held that the ImpFac is a better indicator of the impact of a journal, whereas the H-index is more useful to study the productivity and impact of an individual researcher, we found stronger correlations between the H-index and R and N in this study [30]. Publishing a higher number of papers while maintaining a high citation rate would lead to a high H-index for a journal, and thus, the H-index is probably a better measure of the impact of a journal than the ImpFac. 

The factors that predicted the rate of retraction were different across the different groups in this study. For instance, in anesthesia, none of the variables evaluated (N, ImpFac, H-index) were able to predict the RI. In neurosurgery, the ImpFac predicted the RI whereas for the HICJs, the H-index predicted the RI. Similar discrepancies were noted across groups when multivariable regression was performed to study the possible predictors of the absolute number of retractions (R). In anesthesia, none of the studied variables predicted R. In neurosurgery, ImpFac predicted R whereas for the HICJs, H-index predicted R. These discrepancies could reflect differing publication dynamics across specialties. They could also result from the differing types of papers published across these three groups and the proportions thereof.

## Conclusions

The number of papers published in a journal over a defined period of time increased in proportion to the citation metrics of the journal. As the number of papers published in a journal increased, the number of retractions therefrom also increased. The number of retractions correlated strongly with the citation metrics; more papers were retracted from highly cited journals. The H-index correlated more strongly with the number of retractions than the Impact Factor. There are likely other predictors of the ideal baseline number of retractions as a process-indicator of the pre-publication review process, besides citation metrics and the number of papers published.
